# *In Silico *Evaluation of Predicted Regulatory Interactions in *Arabidopsis thaliana*

**DOI:** 10.1186/1471-2105-10-435

**Published:** 2009-12-21

**Authors:** Damion Nero, Manpreet S Katari, Jonathan Kelfer, Daniel Tranchina, Gloria M Coruzzi

**Affiliations:** 1 Center for Genomics and Systems Biology, Department of Biology, New York University, 100 Washington Square East, 1009 Main Building, New York, NY 10003, USA; 2 Courant Institute of Mathematical Sciences, New York University, New York, NY, 10003, USA

## Abstract

**Background:**

Prediction of transcriptional regulatory mechanisms in *Arabidopsis *has become increasingly critical with the explosion of genomic data now available for both gene expression and gene sequence composition. We have shown in previous work [1], that a combination of correlation measurements and *cis*-regulatory element (CRE) detection methods are effective in predicting targets for candidate transcription factors for specific case studies which were validated. However, to date there has been no quantitative assessment as to which correlation measures or CRE detection methods used alone or in combination are most effective in predicting TF→target relationships on a genome-wide scale.

**Results:**

We tested several widely used methods, based on correlation (Pearson and Spearman Rank correlation) and *cis-*regulatory element (CRE) detection (≥1 CRE or CRE over-representation), to determine which of these methods individually or in combination is the most effective by various measures for making regulatory predictions. To predict the regulatory targets of a transcription factor (TF) of interest, we applied these methods to microarray expression data for genes that were regulated over treatment and control conditions in wild type (WT) plants. Because the chosen data sets included identical experimental conditions used on TF over-expressor or T-DNA knockout plants, we were able to test the TF→target predictions made using microarray data from WT plants, with microarray data from mutant/transgenic plants. For each method, or combination of methods, we computed sensitivity, specificity, positive and negative predictive value and the F-measure of balance between sensitivity and positive predictive value (precision). This analysis revealed that the ≥1 CRE and Spearman correlation (used alone or in combination) were the most balanced CRE detection and correlation methods, respectively with regard to their power to accurately predict regulatory-target interactions.

**Conclusion:**

These findings provide an approach and guidance for researchers interested in predicting transcriptional regulatory mechanisms using microarray data that they generate (or microarray data that is publically available) combined with CRE detection in promoter sequence data.

## Background

Transcriptional regulatory mechanisms have been shown to control metabolic pathways, developmental and cellular processes as well as other functions within the plant as described previously 2-4. Recent work in many eukaryotic species has focused on a Systems Biology approach, using multiple associations between genes, to elucidate regulatory networks and to understand their biological context [5,6]. These associations can be used in combination with gene expression data from microarray experiments and promoter sequence analysis of co-regulated genes, to infer the mechanism for this co-regulation and to search for *cis*-regulatory elements (CREs) that may coordinate this response through transcription factor (TF) activity.

Microarray data analysis can be used to determine sets of genes in the genome that are under coordinate control in response to external treatments [7], or from endogenous signals within the plant such as hormones [8,9]. While this type of analysis can determine the set of genes that are regulated under specific experimental conditions, it does not identify specific *cis *or *trans *acting components involved in this regulation. However, the set of co-regulated genes can be used to identify candidate TF→target relationships using pair-wise associations between TFs and targets based on correlation over microarray data and/or putative CRE detection. This methodology takes advantage of the current data on CRE binding sites for transcription factors as well as current annotation for transcription factors in *Arabidopsis *available in databases such as AGRIS [10]. Using these data in conjunction with pair-wise correlation data allows one to associate TFs with putative co-regulated targets. Previous studies from our group, have shown that analyzing the co-regulation of genes across various experimental conditions in combination with CRE analysis of predicted target gene promoters has been effective in predicting new targets for transcription factors which were then experimentally validated [1,11].

Several currently available database tools including CSB.DB [12], ACT [13], and ATTEDII [14], have used an approach similar to the one described above to predict TF→target relationships to that described above. Specifically, ATTEDII uses microarray data to try to make associations between genes using co-expression alone or correlation in conjunction with CRE analysis. Other tools such as CERMT [15] and ASIDB [16], have focused on using time-course data to identify specific temporal patterns to elucidate transcription factor targets. However, all of these methods rely on a fixed database (of microarrays and CRE elements) and/or analysis format. Consequently, they do not provide a great deal of flexibility for users who may be interested in using their own microarray data, or to adjust the parameters of an analysis (e.g. changing correlation CRE over-representation significance by looking at different p-value cutoffs) for both correlation and CRE methods.

We were thus motivated to develop an approach for predicting regulatory relationships of TF→targets that could exploit microarray data of any design or size, and could encompass any CRE database of interest. Our goal was to test combinations of existing methods for correlation and CRE detection to make predictions for TF→target relationships, which we would then test using microarray data from transgenic/mutant plants for that TF. Our approach is generally applicable for use on genomic sequence and microarray data for plants and any other species for which whole genome data is available.

To evaluate the most effective methods for predicting TF-target pairs, for each microarray data set, we first used *regulated *genes in wild type (WT) plants to predict targets of a particular regulated TF of interest, using both correlation and/or CRE detection methods. We then tested these TF-target predictions using a list of genes shown to be "significantly misregulated (based on ANOVA) in either a TF over-expressor transgenic line or a TF knock-out line. Next, we computed various performance measures including sensitivity, specificity, positive and negative predictive value and the F-measure of balance between sensitivity and positive predictive value. We also tested the performance of several of these measures over *all *of the expressed genes represented on the ATH1 chip - e.g. not only the *regulated *genes.

We believe that our methodology and resulting information will help guide investigators who may have a variety of microarray and CRE datasets and informatic needs for evaluating potential candidates for regulators of responses to various stimuli in plants.

## Results

### Regulatory Predictions using Correlation and *Cis *Regulatory Element (CRE) Analysis

In order to determine putative targets of transcription factors (TFs) in the *Arabidopsis *genome, we have used and combined several existing methods for correlation (Pearson and Spearman Rank (Spearman) correlation) and CRE analysis. Despite the widespread use of correlation methods in the past, we know very little about the performance of these methods (either alone or in combination with CRE detection) in making TF→target predictions, especially using microarray and sequence data. Furthermore, we do not know how these methods compare to each other in making such predictions. Our goal is to compare the performance of these methods and combinations of methods to each other.

Pair-wise correlation analysis, used alone or in conjunction with CRE data, has been shown to be a promising method for predicting TF targets in several studies [1,11,17,18]. Previous studies [13,14] have used Pearson correlation, which indicates the strength and direction of a linear relationship between the expression patterns of two genes over different experimental conditions. Several other correlation methods for making these predictions are also available. Among these are the Spearman's Rank correlation (Spearman correlation) which is a non-parametric correlation measure that is similar to the Pearson correlation, but it is computed on the *ranks *of the data points rather than their measured values. We have evaluated these correlation methods, as they are currently being used for the prediction of linear gene associations using microarray expression data. For each correlation method, a gene is identified as a predicted target, if the p-value of the correlation measure between TF and candidate target is below a cutoff determined by a 5% False Discovery Rate (FDR), (see Methods).

To detect regulatory interactions between TFs and candidate target genes we also used two different methods of CRE analysis, based on a search for known CRE elements within the promoter region of *Arabidopsis *genes (see Methods). The first of these methods computed the frequency in the upstream region of select genes (either from regulated gene lists or from the whole genome) of CRE binding sites that bound to at least one member of a transcription factor family (Note: CRE binding sites were taken from the AGRIS database [10] and manual curation of the literature). Detection of CREs in the promoter DNA Sequence of candidate genes was performed using the RSA Tools DNA Pattern Search Tool [19]. Another method involved determining which CRE binding sites in the upstream region of select genes were at a frequency higher than what would be expected by chance [1,20]. For this, we computed an empirical p-value for the null hypothesis that the CRE frequency observed in a gene of interest is typical of what is seen in the genome as a whole. P-values for each CRE in a given target gene were determined by counting the number of genes in the genome with a CRE frequency greater than or equal to the observed CRE frequency in the given target gene, and then dividing it by the total number of genes in the genome (see Methods). This second approach provided us with a method for narrowing the number of potential TF targets. The motivation for this approach was based on the hypothesis that the greater the number of CRE binding sites, the greater the probability that the TF of interest will bind to one of these sites and activate the gene *in vivo *[21].

Since these two CRE detection methods use experimentally verified links between TFs and their known CRE targets, they provide putative interactions between TFs and Targets containing such CREs in their promoters. Other direct methods for CRE discovery (e.g. novel CRE detection algorithms such as MEME [22]) generally do not provide any data on possible TF interaction. A notable exception to this is CEG (correlation between expression and a defined group of genes) [14], which tries to associate a novel CRE by comparing genes that are correlated with TF expression. However, this method results in candidate CREs that have to be experimentally verified to determine if they are functional.

**Figure 1:**
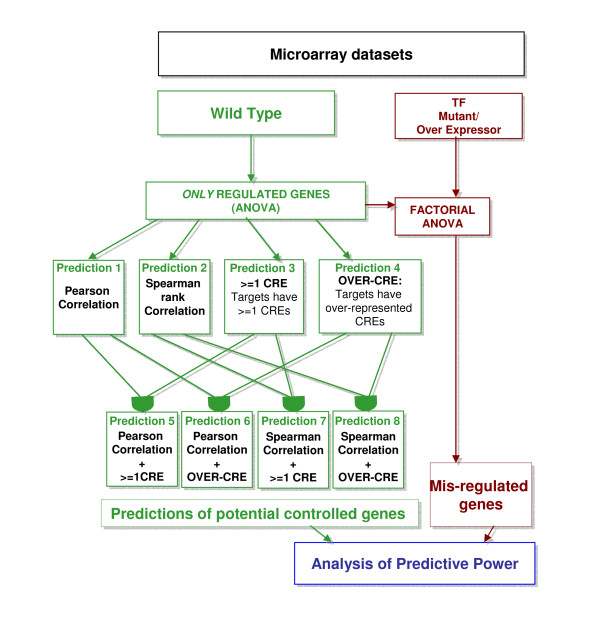
**Conceptual pipeline for TF→target predictions based on correlation and/or CRE analysis: Performed on *Regulated *Genes**. Each data set (3 microarray datasets covering 4 transcription factors, see Table 1) has been subjected to the analysis outlined in Figure 1. For each transcription factor, we predicted 8 different gene lists of potential target genes* using Correlation analysis, CRE detection methods, or the combination of Correlation and CRE analysis methods. The first four lists were predictions made using *only *Correlation or CRE detection methods. The succeeding four gene lists, were generated by the intersection of predicted targets from Correlation *and *CRE methods. Misregulation was determined by comparing wild type microarray data, in which the TF is shown to be regulated, *and *micoarray data from TF mutant/transgenic (transgenic over-expressor). Both data sets contain replicates (at least 2 replicates) from each dataset. Performance was then evaluated using specificity, sensitivity NPV, PPV and the F-measure as statisitcal measures. TFs: Transcription Factors, OVER-CRE: over-represented CRE binding sites, >= 1: greater than equal to 1 CRE binding site *Note that the term *potential target gene *refers to genes that are predicted to be misregulated in the transgenic plants. The analysis does not make any assumption whether this interaction is direct or indirect, or that these predictions are at all inclusive. The possibility that this interaction could be direct is addressed further in the Discussion.

More complex CRE detection methods include identifying combinations of CREs that may be mediating regulatory activities through several TF candidates and use of phylogenetic footprinting techniques to uncover conserved CREs between related species [23,24]. However, these methods introduce many uncontrollable variables such as effective binding site proximity for shared activity, conservation of genome structure between plant species and known regulatory interaction networks, all which have not been extensively characterized in plants. We therefore focused on using known CREs and detection of single binding sites (and/or overrepresentation of CRE sites), as this approach does not involve many assumptions about the regulome and its implementation. Several studies 25-27 have shown that effecting a single CRE in a promoter region is sufficient to alter plant function and we have also shown in previous work [1] that this method is effective in elucidating transcriptional regulators (see discussion for further details). Our proposed methodology therefore stands as an effective and broad based approach that allows for the detection of diverse transcription factors mediating biological responses. The source code for both the correlation and CRE detection tools we have employed in our analysis are available at http://nitro.bio.nyu.edu/regulatoryprediction/. A schema for our overall approach to TF →target predictions based on correlation and CRE, as well as validation is shown in Figures 1 &3

In order to evaluate these correlation and CRE detection methods for their robustness in making correct TF→target predictions over different datasets, we used several publicly available microarray datasets to test their predictive value. These datasets were all processed and evaluated using the same methodology in order to avoid any bias in the analytical methods (see Methods). Datasets were chosen based on their design involving both wild-type (WT) microarray data as well as microarray data for the mutation of a transcription factor under set treatment conditions for both WT and mutant. In our first test, as outlined in Figure 1, all *regulated *genes in WT were considered to be candidate targets of the TF, and only those TFs that were *regulated *under the conditions in which they were treated were used (in order to incorporate correlation prediction methods) in the analysis. Predictions were made using correlation and/or CRE predictions using all of these *regulated *genes, resulting in a subset of putative candidate target genes for the TF. These candidate target predictions were then validated using the mutant/transgenic microarray data for the candidate TF, by determining how many of the predicted targets were misregulated in the mutant/transgenic. We measured the performance of pair-wise correlation and CRE detection methods, by performing correlation and CRE analysis over each individual dataset included in this analysis (see below). The predictions made were for the TF mutagenized in each individual dataset. The overall scheme for this approach to testing and validating methods for TF→target predictions is shown in Figure 1.

### Testing of Regulatory Prediction Methods using Publicly Available Microarray Data

To evaluate the predictive value for TF→target relationships made based on the correlation and CRE methods mentioned above and shown in Figure 1, we chose datasets involving a range of diverse treatment conditions (light, pathogen infection, abiotic stress and hormone treatment) and microarray data resulting from the mutagenesis of diverse transcription factor families, some of which such as ZF-HD and WRKY belong to *plant-specific *transcription factor families, in order to determine a benchmark for the performance of each prediction method in analyzing data from *Arabidopsis thaliana*.

The MIF1 (ZF-HD family) microarray dataset compared the responses of a 35S:MIF1 over-expressor and WT plants to light vs. dark treatment conditions [28]. The ARR7 (MYB-like TF) microarray dataset compared the response of a 35S:ARR7 over-expressor vs. WT to the plant hormone cytokinin treatment [29]. And finally, The WRKY11/17 microarray dataset compared the pathogen response of two redundant T-DNA knockout lines for WRKY 11 and 17 to WT over a time-course experiment comprising four different time points [30]. A summary of the datasets used in this analysis is listed in Table 1. These datasets were chosen based on 3 criteria i) the microarray data was generated using a specific treatment of WT and mutant/transgenic plants with relative controls; ii) replicate experiments for both treated and control conditions in WT and mutant/transgenic treatments; and iii) the transcription factor under study (e.g. transgenic/mutant) was one whose expression is itself *regulated *in WT treatment conditions compared to relative controls.

**Table 1:** Summary of Microarray Datasets Used in this TF→target prediction study.

Transcription Factor Mutant/Transgenic	Treatment	Control	# of Replicate Experiments	Citation
MIF1	Light	Dark	2	Hu et al., (2006)

WRKY11	Pathogen	Water	3	Journot-Catalino (2006)

WRKY17	Pathogen	Water	3	Journot-Catalino (2006)

ARR7	Cytokinin	Water	2	Lee et al., (2006)

Microarray datasets were obtained from publicly available datasets (see Methods for details). This table describes the specific transcription factor being mutagenized in these studies, the type of treatment used in wild-type and mutant/transgenic plants, the control for these treatments, the number of biological replicates in each experiment and the author of the microarray dataset (see reference section for a detailed citation).

Microarray data were processed as described, and differential expression of genes in WT data was determined using ANOVA (see Methods). This approach allowed us the advantage of narrowing down the list of potential candidate genes to only those that were specifically *regulated *over the conditions in each microarray datasets. This list of *regulated *genes also included the TF of interest, which was necessary in order to perform correlation analysis to predict potential targets amongst the *regulated *target genes. The performance measures across these datasets were then evaluated to determine if a consistent pattern for the performance of these regulatory prediction methods could be found across a variety of experimental datasets.

### Evaluation of Regulatory Prediction Methods Using Statistical Tests

To evaluate the performance of these methods for TF→target predictions, we measured the specificity, sensitivity, predictive values (positive and negative) and the F measure (balance between sensitivity and positive predictor value (PPV)) for each of these tests. In the context of our validation study, a gene is said to be a "target of the TF in question (direct or indirect) if that gene is significantly misregulated in the mutant/transgenic TF microarray data (based on ANOVA). Thus, a gene is defined to be a true target if the p-value for the null hypothesis of no change is below a threshold value determined by demanding a nominal false discovery fraction (FDR) of 5% (see Discussion section for limitations of this method). The equations used for these performance measures are described below.

Specificity in this instance is defined as:

Where TN is the number of true negatives (genes that are not misregulated and not predicted to be targets) and FP is the number of false positives (genes that are predicted to be targets but are not misregulated).

Sensitivity is defined as:

Where TP is the number of true positive (genes that are predicted to be targets in the WT and are misregulated in mutant/transgenic microarray data) and FN is the number of genes that are not predicted to be targets but are misregulated in the mutant/transgenic microarray dataset.

And finally, positive (PPV) and negative (NPV) predictor values are defined by the equations  and  respectively, with the aforementioned definition of variables.

As any test can be skewed in favor of the positive predictive value at the expense of sensitivity or vise versa, we also computed the balance between PPV and sensitivity. In order to quantify this balance between these two measures for out various methods of predicting regulatory interactions, we computed a statistical measure borrowed from information retrieval called the F-measure.

The F measure is the harmonic mean of the sensitivity and PPV. It is given by the equation

Where PPV is the positive predictive value and SEN is the sensitivity. The equation for F measure is sometimes stated in terms of *precision *and *recall *which in the current vernacular of bioinformatics and genomics literature are equivalent to PPV and sensitivity, respectively [31].

### Using *Regulated *Gene Lists to Determine the Performance of Regulatory Prediction Methods

**Figure 2:**
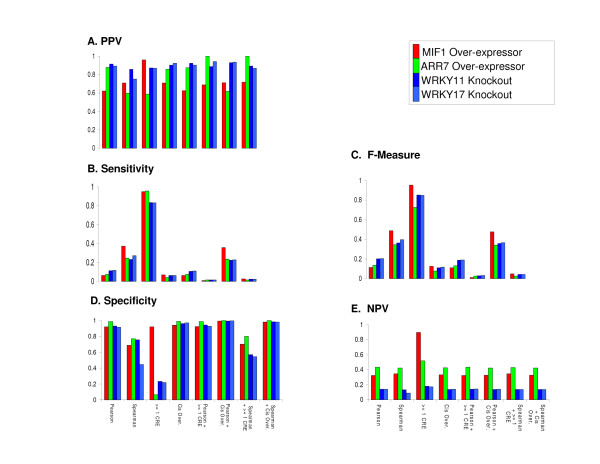
**Performance of Correlation and CRE Prediction Methods across microarray data: *Regulated *genes only**. The overall pattern for the PPV (A), Sensitivity (B) and F-Measure (C), Specificity (D), and NPV (E) for the analysis of regulated genes using correlation and/or CRE detection prediction methods for determining TF targets. As shown here the PPV shows a generally consistent pattern across all datasets, while the sensitivity and F measure are shown to be higher for the Spearman, ≥ 1 CRE and Spearman + ≥ 1 CRE prediction methods (A-C). Also shown is the consistent pattern for specificity and NPV (D and E) across all prediction methods except the ≥ 1 CRE which shows a high degree of variability. PPV (positive predictive value), NPV (negative predictive value), CRE over.: CRE Over-representation, ≥1 CRE: detection of 1 or more CREs.

Using the approaches described above, we analyzed three select datasets of microarray data from wild-type (WT) and three TF mutants/transgenics (MIF1, ARR7, and WRKY11/17) 28-30 to determine how well these prediction methods performed in predicting TF→target relationships (Figure 2). For the MIF1 microarray datasets [28], we found that the Pearson correlation as well as the CRE prediction methods and the combined correlation/CRE methods, had a high specificity for the regulatory target predictions of putative targets of MIF1 (Figure 2D) and a correspondingly low sensitivity (compared to other prediction methods) in most cases (Figure 2B). The PPV was relatively high across all measures (Figure 2A). The NPV was relatively low across almost all measures (Figure 2E). An interesting exception to this, however, is the ≥ 1 CRE method, which has a high performance across all performance measures (including NPV), indicating that this prediction method has a very high probability for success in predicting MIF1 targets (see Additional file 1 Table S1). This result was intriguing and unexpected, as the binding site for MIF1 is the "Core Consensus Sequence (ATTA) (Tan 2006), which is found at a high frequency in promoter regions of the *Arabidopsis *genome, most likely due to the fact that the genome is AT rich in its promoter sequence content [32]. Finding this MIF1 site at such a high frequency, one would expect maximal coverage in capturing a high number of genes that are regulated/misregulated (high sensitivity), but it is a surprising finding that this method also has a high positive predictive value (i.e. a high ratio true positives to false positives (positive predictive value)) (Figure 2A).

It is also noteworthy that the Spearman correlation has a substantially higher F-measure when compared to Pearson correlation with or without the combination of the CRE detection methods, due to its high sensitivity in capturing more misregulated genes than any of the other correlation methods (Figure 2C). The superior performance of the Spearman correlation is most likely due to the fact that the Spearman correlation is a nonparametric correlation measure that does not require a linear relationship between a TF and target gene and therefore is more flexible in capturing the relationship between these genes across expression data.

We find slightly different results in the case of the ARR7 TF microarray dataset [29]. As in MIF1 TF dataset above, we did find a high specificity and positive predictive value for the prediction of ARR7 targets using Pearson and Spearman correlation as well as CRE over-representation and most combined correlation/CRE detection measures and a correspondingly low sensitivity (Figure 2). However, we found that the specificity was much lower in the ≥ 1 CRE method in contrast to the results of the MIF1 data analysis (Figure 2D). Also, we found that the negative predictive value for all methods is consistently higher than seen in other datasets (see Additional file 1 Tables S1 and S3, Figure 2E). This reflects the lower ratio of false negatives (FN) to true negatives (TN), which indicates that in this dataset these tests gave more accurate negative calls. Also, as in the case of the MIF1 dataset, the ≥ 1 CRE method has a high F measure (Figure 2C), due to its high sensitivity (Figure 2B) and PPV (Figure 2A) (and Additional file 1 Table S2) and the Spearman correlation shows a high F measure when compared to other correlation methods (Figure 2C).

One of the CREs used for the analysis of ARR7 was the ARR1AT [33] which binds two component response regulator transcription factors (*Arabidopsis response regulator *or ARR), which is a sub class of MYB-like transcription factors. For this study, we used all experimentally validated binding sites for a TF family in order to best capture the binding relationship between candidate TFs and their and their putative targets, especially in cases where there is no direct information as to which binding site the candidate TF may bind. In the case of all MYB or MYB-like TFs, we used a set of experimentally validated TF binding sites from the AGRIS database and literature curation ([10], see Additional file 1 Table S11 for a list of CRE binding sites) in order to capture the relationship between ARR7 and its putative targets. While this may lead to a greater ambiguity in making predictions it allows for the greatest flexibility in determining putative targets using CRE detection methods.

For the prediction of targets for WRKY11/17 TFs [30] using correlation and CRE detection methods, we used the W-box binding site [34] frequency in order to predict WRKY targets. We found a high specificity for the putative regulatory target predictions of WRKY11 and WRKY17 using Pearson and Spearman correlation methods as well as CRE over-representation (Figure 2). We also found high specificity for most combined correlation/CRE detection measures (with the Spearman correlation having a slightly lower Specificity in the WRKY17 mutant), and a correspondingly low sensitivity and negative predictive value for both of these transcription factors for the Pearson, Spearman, CRE over-representation, and combined prediction methods. We also observe, as in the case of the MIF1 and ARR7 data, that the ≥ 1 CRE method for both WRKY transcription factors has a high sensitivity and a high PPV, giving it a high F Measure (See Additional file 1 Table S3, Figure 2). This is in contrast to its lower specificity which is comparable to the results of the analysis of the ARR7 microarray dataset. We also see a higher F measure in the Spearman correlation when compared to other correlation methods (Figure 2C).

A summary of the PPV (Figure 2A), sensitivity (Figure 2B), F-measure (Figure 2C), specificity (Figure 2D) and NPV (Figure 2E) for all of the datasets analyzed in this study is shown in Figure 2. This figure reveals the consistent pattern of high positive predictive value across all of these datasets for all methods (Figure 2A). This result indicates that these methods tend to capture more true positives than false positives. Figure 2 also highlights the higher sensitivity for the Spearman correlation and ≥ 1 CRE detection methods, separately and combined, when compared to all other methods and their correspondingly higher F-measure (Figure 2C). Further, the plot of the specificity (Figure 2D) for these performance measures, indicates that all prediction methods have a consistent performance pattern across the datasets except the ≥ 1 CRE method. This is most likely due to the variability of direct effects that the CRE may be having, thereby lowering the ratio of false positives to true negatives. And finally, the NPV (Figure 2E) shows a consistent pattern across all datasets, except again in the case of the ≥ 1 CRE method - due to the lower ratio of false negatives to true negatives.

### Evaluation of Spearman Correlation +/- CRE Detection Regulatory Prediction Methods over the Entire ATH1 Microarray Chip

**Figure 3:**
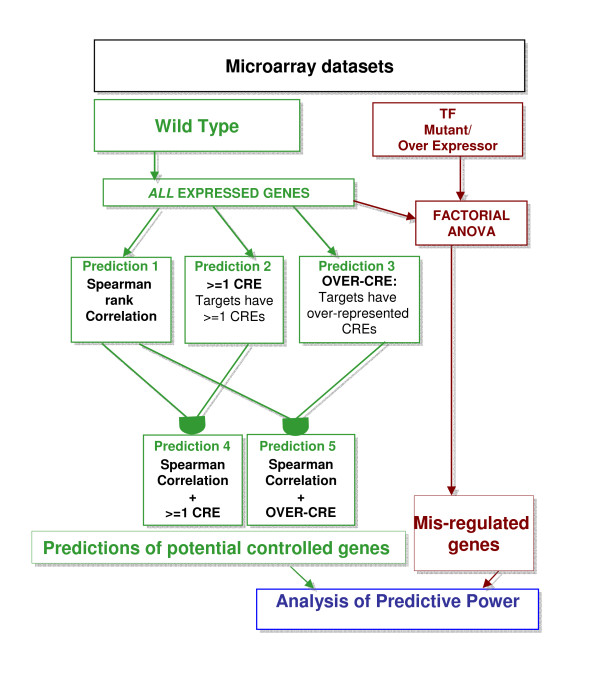
**Conceptual pipeline for TF→target predictions based on correlation and/or CRE analysis: Performed on *All Expressed *Genes**. The strategy for determining TF→target interactions using both correlation analysis and CRE detection methods, using all *expressed *genes with a "Present call on the Affymetrix ATH1 microarray chip using the methodology previously described (see Figure 1). The analysis was reduced to using the Spearman correlation (which was previously shown to be the have more balanced performance compared to other correlation methods (based on the F measure)) alone or combined with CRE detection methods in order to determine changes in performance for these measures over the entire genome. TFs = Transcription Factors

One surprising result of the above analysis of *regulated *genes was the outstanding performance of the ≥ 1 CRE method. While one would expect this method to have a high sensitivity, one would expect this method to have a low specificity and low PPV due to the potential for false positives. However, the ≥ 1 CRE method had a high PPV (Figure 2A) across all datasets and it also had a high specificity in the MIF1 dataset (Figure 2D). The excellent performance of the ≥ 1 CRE method may stem for our using only *regulated *genes for this analysis; this may have created an artificially high specificity and PPV due to the fact that many *regulated *genes are also *misregulated *in the mutant/transgenic. Thus, if a gene has the CRE binding site, it has a greater chance of being a TF→target, as it is *regulated *under the same conditions as its putative regulator (TF). To test this hypothesis, we made regulatory predictions using the ≥ 1 CRE for the candidate TFs in each dataset across *all *expressed genes represented on the ATH1 microarray chip (e.g. not *only *the *regulated *genes) (See analysis scheme in Figure 3).

We also wished to further test the Spearman correlation on this new dataset (*all *expressed genes on the ATH1 chip) as we found Spearman correlation to be the most balanced method (based on F measure) for capturing putative targets among regulated genes we hypothesized that it might have the most robust performance over the entire genome. To test this, we performed Spearman correlation between the candidate TF from each dataset and *all *the genes represented on the ATH1 microarray chip and expressed in the microarray data. This was done in conjunction with CRE detection methods performed over the entire genome, in order to determine how well the CRE detection methods performed on their own vs. combining the Spearman correlation and CRE detection methods. By combining these methods over the whole genome, we may improve the performance of these methods in capturing putative target genes.

**Figure 4:**
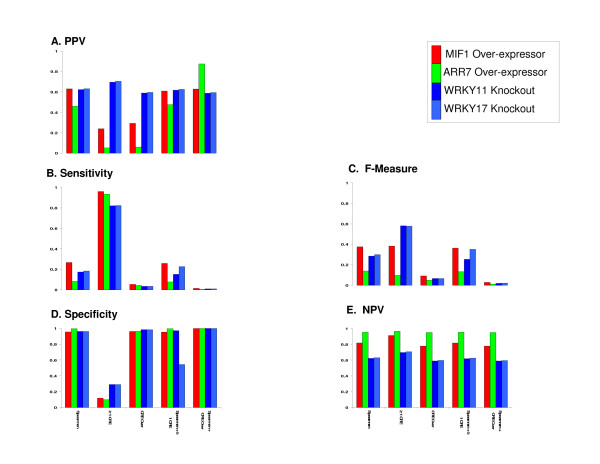
**Performance of Correlation and CRE Prediction Methods across microarray data: *All Expressed Genes *(Whole Genome Prediction Analysis)**. The overall pattern for the PPV (A), Sensitivity (B) and F-Measure (C), Specificity (D), and NPV (E) for the analysis of all genes represented on the ATH1 microarray chip using correlation and/or CRE detection prediction methods for determining TF targets. The results of this analysis show a more highly variable PPV (A) for both CRE detection methods and a consistent PPV for all methods involving the Spearman correlation. Also shown is a reduced and far more variable F measure for the ≥ 1 CRE method indicating a loss of consistently strong performance in balance between the sensitivity and PPV. This loss of performance is also seen in the consistently low specificity (D). The NPV (E) shows a consistent pattern of response across all measures. One interesting observation is that the Spearman correlation combined with CRE over representation has the highest and most consistent PPV and specificity across all datasets for this analysis, comparable to what was seen in the previous analysis (Figure 2). CRE Over: CRE Over-representation, ≥1 CRE: detection of 1 or more CREs, Spearman: Spearman Rank Correlation.

A summary for the results of this analysis of the correlation and CRE methods used on all *expressed *genes detected with the ATH1 chip is given in Figure 4, as well as in Additional file 1 Tables S4-6. The histogram plots in Figure 4 show the PPV, sensitivity and F-measure, specificity and NPV for all of the datasets analyzed in this study. Figure 4A shows that the PPV is consistent across datasets for the Spearman correlation and the combined CRE/correlation methods. However there is a high degree of variability in the PPV measure for the CRE method (Figure 4A). Figure 4A also reveals a consistent pattern of PPV across all of these datasets for the combination of Spearman correlation and CRE over-representation. Figure 4B shows the sensitivity, which is low for all prediction methods except ≥ 1 CRE due to the high coverage across the genome for these binding sties. Figure 4C shows the F measure, which is much more variable in the ≥ 1 CRE method vs. the Spearman or Spearman + ≥ 1 CRE and low for all other measures. The high F measure seen in the WRKY11 and WRKY17 predictions is most likely due to the high number of genes found (9,445 and 9,308 respectively) to be *expressed *across the genome in these microarray samples, giving a higher coverage of the ATH1 genome chip than was found with other datasets. Figure 4D shows a consistently high specificity for all prediction methods except for the ≥ 1 CRE, which is consistently low. Finally, and Figure 4E shows a consistent pattern of NPV across all measures. In all, these results confirm that the ≥ 1 CRE performance diminishes when this method is applied to all expressed genes (Figure 4) (vs. *regulated *genes shown in Figure 2), based on the variable PPV and F measure, and the consistently low specificity in the *expressed gene *analysis (Figure 4).

### Evaluating the Algorithm for Computing Correlations

As indicated in our results, the Spearman correlation gives the best balance in terms of having a high PPV and a higher sensitivity than other correlation methods (Figure 2). The underlying theory for this is that as the Spearman correlation is a rank based measure of pair-wise association and as such the Spearman correlation does not require a linear relationship between the expression of a TF and a candidate target gene. To demonstrate this idea, we took the microarray data from each dataset for those genes predicted to be targets of a specific TF (as described above) and squared the expression values for these genes in each individual dataset. This new dataset was then combined with the original expression values for the TF in question and the correlation was performed again using the Spearman and Pearson correlation. Because the squaring transformation corrupts existing linear relationships between TF and target expression levels but has no effect on ranks, we expect degradation in performance of the Pearson method but no change in that of the Spearman method.

Based on our results (see Additional file 1 Tables S7-S10), we see that indeed the Spearman correlation is unaffected while the Pearson correlation is markedly reduced in the number of predicted targets (in some cases not having any predicted targets). These results clearly demonstrate the differences in the correlation methods and their behavior fits well with the previously described assumptions made in the correlation methods.

The superior performance of the Spearman correlation method over Pearson correlation above is consistent with previous evaluations of these methods [35,36]. Rank correlations are not easily influenced by skewness or the high levels of variability that is often observed in Affymetrix microarray data of the type that was used in this study. In contrast, the Pearson correlation method is very sensitive to noise in the data due to extreme values, improper normalization and skewness.

## Discussion

In this study, we have used several correlation methods (Pearson and Spearman correlation), as well as CRE detection methods (≥1 CRE and CRE Over-representation), in order to predict targets of regulated genes in wild-type that may be confirmed to be targets as genes that are misregulated using microarray data from a TF mutant/transgenic. The purpose of using and combining these methods was to attempt to make predictions for associations between candidate TFs and their targets using microarray expression data and sequence data. While similar approaches have been used before in several studies [13,14], to date correlation and CRE detection methods have not been compared previously to determine which is the most effective (alone or in combination) in a practical application on microarray data from datasets of wild-type and TF mutants for Arabidopsis genomic data. Further, no current tool allows for the flexibility of using ones *own *microarray data, as well as several different CRE and correlation based methods (as well as the overlap for predicted targets), in order to make predictions of putative TF →target relationships. In order to make this assessment, we used several datasets, taken from the literature for canonical examples of microarray expression datasets involving TFs regulated by several different types of external stimuli (e.g. light, pathogen infection, hormone response and abiotic stress response) that represent several TF families, including two plant-specific TF families. The mutagenesis of these TFs within these microarray studies then allowed us to evaluate how well our prediction methods captured genes that would be affected by candidate TFs on a transcriptome level.

We assessed the performance of various methods of predicting TF targets over datasets by computing several statistical measures: specificity, sensitivity, PPV, NPV and F measure (Figures 2 &4). We feel that the results of this assessment provides a useful resource for researchers performing genomic research using microarray datasets who wish to elucidate transcriptional regulatory mechanisms controlled by transcription factors of interest. And while we have applied this approach specifically to *Arabidopsis*, these tools are applicable to genomic data from any species.

### Analysis of Correlation Methods for TF Target Predictions Yields Two Approaches for Determining Target Genes of Interest

We found that for *regulated *gene lists (e.g. genes significantly regulated in response to a specific treatment), we have shown that the Spearman correlation method gave the most balanced predictions for TF targets (based on the F measure) (Figure 2). By contrast, we found that the Pearson correlation method provided greater specificity than the Spearman correlation in all of the datasets analyzed (Figure 2). This result provides two perspectives as to the most effective way to capture genes of interest, given an experimental design. While some investigators' experiments are focused on capturing a large number of genes, others are interested in capturing specific groups of genes that may belong to a functional group or pathway of interest or may be unique in simply sharing a pattern of regulation. Thus, the Pearson correlation is better suited to smaller lists of genes for empirical validation (by Quantitative PCR, for example), while Spearman correlation is better suited to large scale analysis covering many genes. These two correlation methods therefore provide an effective means for generating hypothesis for various experimental designs.

### Analysis of CRE Detection Methods reveals the ≥ 1 CRE Performance Varies Given the Gene List being analyzed

For CRE detection methods, presence of ≥ 1 CRE yielded excellent performance results when applied to only those genes that are *regulated *under the experimental conditions of these datasets (Figure 2). Although this result initially seemed counterintuitive to us, comparing the results based on *regulated *genes to those based on *expressed *(but not necessarily regulated) genes represented on the ATH1 microarray chip gave insight as to the cause of this high performance of the CRE method. Indeed, we saw a remarkable drop in both the specificity and PPV for ≥ 1 CRE method for regulatory predictions made by using *all expressed genes *vs. those made by using only *regulated *genes (Figure 2) (Figure 4) on the ATH1 microarray chip. This is most likely due to an artificially high PPV that is introduced when looking at *only regulated *genes (Figure 2).

As the microarray datasets used in this study involved TFs whose expression is *regulated *in response to the treatment, the treatment conditions were those in which these TFs were expected to be regulated, and therefore be good candidates for controlling the response of target genes that were also responsive under these treatment conditions. Thus, there is a high probability for genes that are *regulated *to also be *misregulated *in a mutant/transgenic that is specifically responsive under the treatment conditions being studied. This, coupled with the fact that the CRE binding sites used in this study are found in very high frequency across the genome, gave good odds of finding a "true target vs. generating a false positive. However, when taken out of the context of looking at only *regulated *genes the ≥ 1 CRE performs poorly (Figure 4), as expected, as many false positives were generated due to the large number of CRE matches that are found across the entire genome.

### CRE Over-representation Yields Evidence of the Effectiveness of Combining Correlation and CRE Regulatory Prediction Methods

As shown for the results of the ≥ 1 CRE regulatory prediction method, the performance of the CRE over-representation regulatory prediction method was also very dependent on the gene list used. For this method we also observed a loss of specificity and PPV when it was applied to the entire list of ATH1 microarray chip genes vs. only those genes regulated under the experimental conditions of each dataset (Figure 4). This is likely due in part to the fact that extending this and other methods to all expressed genes increases the number of possible matches. This in turn alters the significance of the p-values generated because the FDR cut-off used in this study is subject to the distribution of p-values.

However, we see a notable improvement in the PPV when the CRE over-representation method is combined with the Spearman correlation and applied to all *expressed *genes (Figure 4). Indeed, combining the Spearman correlation with CRE over-representation sometimes gave a substantially higher performance than what was seen with either of these methods alone.

### Limitations of Using CRE Detection Methods

We mention several caveats in using this methodology to make regulatory predictions in *Arabidopsis*. One involves the CRE detection methods used. A major limitation of the CRE approaches described in this work is that they rely heavily on the existence of a known CRE binding site for a individual candidate TF or for a well characterized family or subfamily of TFs. This type of data is extremely limited in *Arabidopsis*. More work needs to be done in order to verify TF→target interactions and in both annotating and discovering CRE and TF function. Another limitation is that not all transcriptional control of gene regulation is based solely on the function of transcription factors. Indeed, while it is true that a substantial amount of transcriptional regulation can be accounted for by transcription factors [37], several additional mechanisms exist that could account for this control of gene expression on a transcriptome level including microRNAs [38] and chromatin modification [39]. Nevertheless, based on these results it seems that presence of ≥ 1 CRE and CRE over-representation are useful tools in making regulatory predictions for targets of TFs when the data on the TF and the CRE binding site are available and when the TF and target mRNAs are *regulated *in response to treatment.

### Limitations of Regulatory Prediction Methods

Our overall schema to predict regulatory interactions between transcription factors and putative targets in the *Arabidopsis *genome (Figure 1) has several limitations due to the complex nature of gene regulatory interactions. Not only are transcription factors able to regulate themselves, but there are temporal relationships between the expression of transcription factors and their targets, a phenomenon which has been addressed by other tools [15]. Further complicating this is the existence of feedback [40], feed-forward [41] and other complex network motifs within the *Arabidopsis *regulome that may be affecting the interaction between transcription factor regulators and their targets.

Thus we acknowledge that our approach, which seeks to make direct relationships between TFs and targets, may be missing some of the complexity of the regulatory interactions that exist within the *Arabidopsis *genome. Our implicit assumption is that differential expression of a gene between WT and a TF mutant/transgenic is a result of a direct regulatory interaction between the TF and the target. However the interaction may be indirect or even compensation from a parallel regulatory pathway. Furthermore, our correlation analysis can only capture those TF-target interactions that are tightly co-regulated. Similarly, by using only the CRE methods, we are limited to only those TFs that directly bind their targets and do not act through intermediate transcriptional regulatory mechanisms. We feel however that our approach still has a great deal of utility, as it allows for a method for hypothesis generation and testing when evaluating potential TF candidates from mutant/transgenic TF studies, as has been shown in one of our recent publications [1].

### Comparison to Other Regulatory Prediction Methods

Several methods currently use correlation and CRE search methods in order to predict regulatory interactions between TFs and putative targets. Examples are CERMT (Covariance-based Extraction of Regulatory targets using Multiple Time series) [15], which predict targets for a particular transcription factor of interest. Targets are defined as genes that respond similarly to a transcription factor, with or without a time shift. ATTED II [14] uses a fixed database of 58 experiments (1,338 slides) and identifies CREs located 200bp upstream of a gene and a transcription factor that are correlated across the expression data set. While these methods are effective, they are limited by their use of either a fixed database or of a specific data type.

In the case of CERMT [15], the authors state that the program is only effective on time course microarray data. ATTED II [14] relies on a fixed microarray database for correlation and requires that one use a fixed upstream region for CRE discovery. Our approach differs in that it can be used with any properly formatted genomic data. Correlation is computed on any microarray data that is formatted into a data matrix, which is then used to identify putative TF-target interaction based on correlation alone. The presence or over-representation of known CREs found in the promoter region of a putative target is computed and used to create putative TF-target interactions based on CRE alone. The results of the two methods are compared to determine the overlap between CRE and correlation TF-target predictions. Our methods and approach have been implemented in the VirtualPlant software platform which is currently available online at http://www.virtualplant.org.

### Validation of Approach of TF→target predictions in the 35S:CCA1 Over-expressor Study

The approach of using a combination of correlation and CRE detection methods was recently found to be successful in our predictions and validations [1] involving the identification of a nitrogen response network controlled by the CCA1 transcription factor. The purpose of that study was to use a genomic approach to identify gene networks whose expression is regulated by nitrogen and/or glutamate-derived metabolites in plants. Specifically, the investigators were interested in the regulation of the nitrogen assimilation pathway in response to nitrogen signaling.

This is a critical pathway that is necessary for nitrogen uptake and storage whose components have been shown to be a strong indicator of transcriptional response to nitrogen in plants [7,42]

The results of that analysis resulted in 834 genes that were shown to differentially respond to various inorganic (ammonium nitrate) and organic (glutamate or glutamine) nitrogen treatments.

In order to determine what genes may be regulating this response to nitrogen, a combination of Pearson correlation and over-representation of CREs was used. This was done in order to narrow down the list of targets to specific pathways involved in nitrogen assimilation. Based on these prediction methods, several targets were predicted to be regulated by CCA1 including ASN1, GDH1 and GLN1.3, all of which are present within the nitrogen assimilation pathway.

Using an over-expressor line for CCA1 (35S::CCA1), the investigators in Gutierrez et al (2008) [1] were able to show that the predicted TF→target genes were indeed regulated by CCA1 based on quantitative PCR results showing the misregulation of these genes in the 35S::CCA1 over-expressor. These results demonstrate the effectiveness of these methods (correlation and CRE representation) in predicting TF→target relationships using large-scale transcriptional changes.

## Conclusions

The study presented here illustrates the effectiveness of using correlation and CRE detection methods alone or in combination with correlation methods, in order to predict targets of candidate TFs. We have been able to show both the effectiveness of these methods overall in capturing regulated predicted targets that are also misregulated in TF mutants/transgenics. We also compared different correlation and CRE detection methods to determine which are the most effective by various measures of performance over various microarray datasets. These tools provide a valuable resource in effectively predicting transcription factors controlling transcriptional responses on a genomic level.

## Methods

### Microarray Dataset Processing

Published ATH1 Affymetrix microarray datasets were taken from the NASCarrays NASC microarray database (Nottingham *Arabidopsis *Stock Center [43] and ArrayExpress [44] databases or requested directly from the author (MIF1 data set [21]). Only microarray data with at least 2 biological replicate experiments were used in this study (see Table 1 for the number of replicates in each dataset). Genes with Absent/Marginal calls had their signal values replaced by a non numerical NA placeholder which is ignored by the algorithms used in this study. All microarray data was processed and normalized using the R MAS5 method with global signal intensities scaled to an average signal intensity of 100 and all other values for this function set to their defaults.

### ANOVA of Microarray Data to Determine Regulation/Misregulation

One way ANOVA was performed on the treated vs. control experiments in WT in order to determine differentially regulated genes. All time courses were treated as separate experiments and were compared to their relative controls. Genes that changed at any time point were considered to be differentially expressed. To determine misregulation in mutant/transgenics compared to wildtype experiments, Factorial ANOVA was performed in R using the following model Y = μ + αTreatment + βGenotype + γGenotype: Treatment, where *Y *is the normalized MAS5 expression signal of a gene; μ is the global mean; and αcoefficients correspond to the effects of the different factor levels Treatment and Genotype. All genes differentially regulated using this formula are considered to be misregulated.

### Computation of Spearman Rank Correlation

Spearman correlation predictions were computed using the MATPACK function available in C++ library http://www.matpack.de/. This function computes and tests the pair-wise associations between items using a correlation coefficient. P-values for the correlation are derived from the correlation coefficient generated for each pair-wise correlation. A 5% FDR cutoff was used to determine those p-values that were significant.

### Computation of Pearson Correlation

Pearson correlation predictions were computed using a C++ based program using the methodology described in Opgen-Rhein et al., 2006 [45]. C++ was used as correlation analysis is computationally intensive, especially for large numbers of genes. Boost http://www.boost.org, a widely used set of C++ templates, was used to construct matrices from expression data. For each TF-putative target gene predicted by the Pearson correlation, the p-value for their interaction was determined by the probability of observing a correlation as large or larger under the null hypothesis of no true correlation. This p-value was estimated by independently randomizing the experiment labels on the expression values of each gene and computing the correlation matrix for each randomization. A 5% FDR cutoff was used to determine those p-values that were significant.

### CRE Search (≥ 1 CRE)

CRE matches to known CREs were generated using the DNA pattern search tool available from RSA tools [19] with a maximum of 3,000 bp of the upstream promoter sequence of candidate genes being searched in the forward and reverse direction for matches to known CREs. Only genes present on the *Arabidopsis *ATH1 gene chip were used in order to facilitate comparison and overlap with correlation results. Known CREs for a TF of interest were obtained from the AGRIS database [10] or from manual literature curation. Promoter sequences for target genes were downloaded from parsed promoter sequence for the *Arabidopsis *genome available from the AGRIS database [10].

### CRE Over-representation

Over-representation of *cis *regulatory elements (CREs) was determined by using an empirical p-value to compute the probability for a CRE corresponding to a transcription factor of interest. This p-value was computed by taking the number of genes in the genome with a frequency greater than or equal to the observed frequency for a given gene divided by the total number of genes in the genome. Only genes present on the *Arabidopsis *ATH1 gene chip were used in order to facilitate comparison and overlap with correlation results.

### Computing the False Discovery Rate (FDR)

The FDR or the expected fraction of false positives among all genes reported as significant was used to determine a significance cutoff for all p-values used in this study. The FDR was computed in R using the Storey and Tibshirani (2003) [46] method. This method determines a cut-off based on the expected proportion of false positives incurred when calling that feature significant.

## Authors' contributions

DN carried out the primary analysis, developed study design and drafted the manuscript. MK contributed to software implementation and to the study design. JK wrote C++ code for the software. DT contributed to the study design, statistical analysis and software implementation. GM conceived study and participated in its design and coordination. All authors read and approved the final manuscript.

## Supplementary Material

Additional file 1 **Supplementary file**. Supplementary tables 1-11 Click here for file
